# In vitro antioxidant and antitumor study of zein/SHA nanoparticles loaded with resveratrol

**DOI:** 10.1002/fsn3.2302

**Published:** 2021-05-07

**Authors:** Qiankun Shi, Xinya Wang, Xudong Tang, Nuo Zhen, Yupeng Wang, Zhijian Luo, Hao Zhang, Jingsheng Liu, Dongfang Zhou, Keke Huang

**Affiliations:** ^1^ College of Food Science and Engineering National Engineering Laboratory for Wheat and Corn Deep Processing Jilin Agricultural University Changchun China; ^2^ School of Pharmaceutical Sciences Southern Medical University Guangzhou China; ^3^ Department of Food Science Rutgers University New Brunswick NJ USA; ^4^ State Key Laboratory of Inorganic Synthesis and Preparative Chemistry College of Chemistry Jilin University Changchun China

**Keywords:** anticancer activity, antioxidant activity, nanoparticles, resveratrol, SHA, zein

## Abstract

Resveratrol (RES) loaded Zein‐SHA (low‐molecular‐weight sodium hyaluronate) nanoparticles with average diameter of about 152.13 nm and polydispersity index (PDI) of 0.122, which can be used to encapsulate, protect and deliver resveratrol. By measuring ABTS free radical scavenging ability and iron (III) reducing power, it was determined that encapsulated resveratrol has higher in vitro antioxidant activity than free resveratrol. When tested with murine breast cancer cells 4T1, the encapsulated resveratrol also showed higher antiproliferative activity than free resveratrol, with IC_50_ values of 14.73 and 17.84 μg/ml, respectively. The colloidal form of resveratrol developed in this research may be particularly suitable for functional foods and beverages, as well as dietary supplements and pharmaceutical products.

## Graphical abstract

Resveratrol nanoparticles with zein as carrier were successfully prepared. RES nanoparticles have better in vitro antioxidant activity and antitumor effect than free resveratrol. RES could be a natural compound for development of nutraceutical and cosmeceutical products.

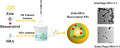

## 1 INTRODUCTION

Resveratrol (RES) is a natural polyphenol molecule with anti‐inflammatory (Gambini et al., 2015; Pujara et al., 2021; Vella et al., 2015; Wahab et al., 2017), antioxidant (Schlich et al., 2020; Wang, Zhao, et al., 2020), anticancer (Chaudhary et al., 2019; Lin et al., 2020; Liu et al., 2020; Yin et al., 2020), hepatoprotection (Lee et al., 2012) effects. In addition, RES can improve metabolic disorders in diabetic rats (Liohanna et al., 2020). RES also has antiobesity effects, that could significantly improve metabolic phenotype and intestinal oxidative stress in the high‐fat diet‐fed mice (Wang, Zhao, et al., 2020; Wang, Gao, et al., 2020). Although resveratrol has many beneficial benefits to the human body, it still has its own shortcomings such as poor solubility, low bioavailability, and unstable chemical properties, which limit its application in food, nutrition, and medicine. In order to break these limitations, delivery systems are designed and applied. Among the delivery systems, nanocomplexes have been widely employed in food fields relying on their advantages, such as effective targeting and sustained release (Guo et al., 2020).

A variety of nanoparticle‐based systems have been investigated for their potential to encapsulate, protect, and deliver resveratrol. Previous studies have used various kinds of nanocarriers to encapsulate resveratrol, including hydrophobic proteins (Iris et al., 2015), cyclodextrin metal‐organic framework/chitosan (CD‐MOF/CS) nanocapsules (Qiu et al., 2020). Liposomes (Gambini et al., 2015), alginate‐CaCl_2_ microspheres (Cho et al., 2014), cyclodextrins (Kaur et al., 2015), Gelatin (Karthikeyan et al., 2015), bovine serum albumin (Guo et al., 2015), silk protein (Lozano et al., 2014), and zein (Penalva et al., 2015).

Among these, zein has been widely studied. Zein encapsulated tea polyphenols nanocarriers show good performance against external environmental stress and protect tea polyphenols from damage (Ba et al., 2020). Zein nanocarriers can significantly improve the photostability of lutein (Frankjen et al., 2020).

Zein has a special amino acid composition, it is rich in glutamic acid (21%–26%), leucine (20%), proline (10%) and alanine (10%) that are hydrophobic amino acids, therefore it cannot be dissolved in water and has unique self‐assembly characteristics. Zein based nanoparticle protects resveratrol from isomerization by UV light (Cheng et al., 2020). Zein in addition to work as a carrier, it also can work to boost the better antioxidant activity than free ingredients (Shi et al., 2020).

In the current research, we used the antisolvent coprecipitation method to prepare Zein‐SHA nanoparticles. Sodium hyaluronate can be complexed with zein, which can increase the stability of the nanoparticles in aqueous solution, and the nanoparticles have pH Response characteristics. We used the approach to fabricate resveratrol‐loaded nanoparticles, and then compared their in vitro antioxidant and anticancer activities with free resveratrol. The ultimate aim was to show that these nanoparticles could be used to encapsulate resveratrol in a form that could be successfully utilized in functional food, nutrition, and pharmaceutical products.

## 2 MATERIALS AND METHODS

### 2.1 Materials

Zein (98%) were purchased from J&K Scientific (China), 2, 2′‐Azino‐bis (3‐ethyl‐ benzothiazoline‐6‐sulfonic‐acid) diammonium salt (ABTS, >98%) were purchased from Energy (China), thiazole Blue (MTT) and resveratrol (>98%) were purchased from Sigma‐Aldrich. Low‐molecular‐weight sodium hyaluronate was purchased from Freda (48 kDa). Other chemical reagents were of analytic grade.

### 2.2 Solution preparation

#### 2.2.1 Purification of zein

Zein dissolved in DMSO (0.2 g/ml). The solution was then settled in dichloroethane at a ratio of 1–10 (v/v), repeated three times. Then dip with petroleum ether and filter with suction three times, and left it in a vacuum oven to dry it to obtain white powder.

#### 2.2.2 Zein solution

Dissolved zein in 90% (v/v) ethanol aqueous solution to prepare 2.5% (w/v) zein solution, then put in 4°C refrigerator for use.

#### 2.2.3 Resveratrol‐zein solution

5 mg of RES dissolved in 2.5 mg/ml zein solution, magnetically stir for 10 min, put in 4°C refrigerator for use

#### 2.2.4 SHA solution

Low‐molecular‐weight sodium hyaluronate powder dissolved in double‐distilled water, magnetically stir for 10 min, perform ultrasonication for 30 s to fully dissolve it.

### 2.3 Preparation of resveratrol‐loaded Zein‐SHA nanoparticles

Added the prepared RES‐zein dropwise to the SHA solution in a ratio of 1:4, kept stirring at 700 rpm, and then removed the ethanol through dialysis (MWCO, 3,500 Da) (Wang et al., 2017).

### 2.4 Nanoparticle characterization

#### 2.4.1 Particle size and zeta potential measurements

The average particle size, polydispersity index, and zeta potential of freshly prepared nanoparticles solution(pH = 5.0) were measured at 25°C using DLS (Brookhaven 90 Plus).

#### 2.4.2 X‐ray diffraction

Used X‐ray CCD single crystal diffractometer to measure RES‐ZEIN‐SHA NPs, proportional physical mixture and X‐ray diffraction (XRD) patterns of three components. The XRD patterns were recorded at a scanning range of 10°–40° (2θ) and a scanning rate of 5°/min.

#### 2.4.3 Transmission electron microscope

A certain concentration of nanoparticles solution was dropped on a carbon‐coated copper mesh, dried by natural air, and the morphology of the nanoparticles was observed and analyzed by Transmission electron microscope (JEM‐1011, JEOL).

#### 2.4.4 Resveratrol determination

An ultraviolet spectrophotometer was used to determine the content of resveratrol in the nanoparticles. An aliquot of freeze‐dried nanoparticles (20 mg) was dissolved in 10 ml DMSO, and stirred for 2 hr in the dark. Then the solution was centrifuged at 13,700 *g* for 30 min. The supernatant was diluted with DMSO and analyzed by UV‐visible spectroscopy at 327 nm, and the content of resveratrol was calculated by the standard curve, which was established using a standard solution in the range of 0–10 μg/ml (*R* ^2^ = .9977).

#### 2.4.5 Particle yield and resveratrol‐loading efficiency

The freshly prepared colloidal dispersion was dialyzed to remove free resveratrol and organic reagents, and then centrifuged at 1,200 *g* for 5 min to remove large particles, and then freeze‐dried. Particle yield and resveratrol loading use formula to determine efficiency were determined using equations (Equation 1) and (Equation 2):

(1) $$\text{Particle yield}\left(\text{\%}\;\right)=\left(\frac{\text{weight of the freeze dried nanoparticles}}{\text{total weight of RES},\;\text{Zein and SHA}}\right)\times 100\text{\%}\;$$

(2) $$\text{Loading efficiency}\left(\text{\%}\;\right)=\left(\frac{\text{RES in nanoparticles}}{\text{total RES input}}\right)\times 100\text{\%}\;.$$

#### 2.4.6 In vitro resveratrol release

Measured the release of resveratrol in vitro using the previously reported dialysis method. (Wang et al., 2018). Put 2 ml of RES loaded zein‐SHA NPs with a certain concentration into the dialysis bag. The dialysis bag was completely immersed in the dialysate (PBS solution, pH = 5.0, pH = 7.4), and the measurement was carried out at 37°C and 100 rpm. 1 ml of dialysate was taken out at certain intervals, and 1 ml of corresponding fresh buffer was added, and the content of resveratrol was measured by UV. The sampling points were 1, 2, 4, 6, 10, 12, 24 hr.

#### 2.4.7 Stability studies

In order to determine the storage stability of the nanoparticles, the nanoparticles were stored in the dark at 25°C for 14 days, and the stability was evaluated by measuring the average size, PDI and potential of the nanoparticles. Stability experiment repeated three times.

### 2.5 Antioxidant activity measurements

#### 2.5.1 Determination of ABTS free radical scavenging ability

The ABTA radical scavenging ability was detected with reference to previous methods (May et al., 2015; Zhang et al., 2020). In order to obtain the ABTS radical solution, 7 mM ABTS solution and 4.9 mM potassium persulfate solution were mixed in equal volume for 12 hr under dark conditions. The absorbance of ABTS radical diluted with PBS buffer at 734 nm was 0.80 (±0.02). 0.5 ml of different samples were mixed with 3.5 ml of ABTS free radical solution and stabilized for 5 min in the dark, and the absorbance at 734 nm was measured. The PBS buffer used to disperse nanoparticles and the absolute ethanol used to dissolve resveratrol were used as blank controls, and the clearance rate was calculated by the following formula (Equation 3):

(3) $$\text{scavenging}\;\text{percentage}\left(\text{\%}\;\right)=\left(\frac{A_{0}‐A_{1}}{A_{0}}\right)\times 100\text{\%}\;$$

A_0_ was the blank control, A_1_ was the absorbance of the sample.

#### 2.5.2 Determination of reducing power

The previous method was used to determine the reducing power of the sample (Gulcin, 2006; Huang et al., 2017). Different concentrations of resveratrol nanoparticles in distilled water or pure resveratrol dissolved in ethanol were added to a mixture of PBS (5 ml) and potassium ferricyanide (5 ml, 1%, w/v). The solution was incubated in 50°C water solution for 20 min, then cooled to room temperature. After, 2.5 ml of the resulting solution was mixed with distilled water (2.5 ml), reacted with FeCl_3_ solution (0.5 ml, 0.1%, w/v) for 10 min, and then measured at 700 nm in a spectrophotometer. All samples were carefully protected from light throughout the experimental procedure.

### 2.6 Cell culture and anticancer assay

The MTT method was used to detect the toxicity of samples to mouse breast cancer cells (4T1). 1 × 10^5^ cells were sown in a 96‐well plate containing DMEM medium and cultured in a 37°C, 5% carbon dioxide incubator. After 12 hr, added the medium for dissolving different samples to the 96‐well plate. After 24 hr, added MTT (5 mg/ml in PBS) and incubate for 4 hr. Then removed the medium, added 150 ml of DMSO, incubated for 10 min, used a microplate reader to measure the absorbance at 490 nm, and set three replicate wells for each concentration. Calculated cell viability according to the following formula (Equation 4):

(4) $$\text{Cell}\;\text{viability}\;\left(\%\right)\;=\;\left(\frac{A_{t}}{A_{c}}\right)\;\times \;100\%$$

*A*_t_ and *A*_c_ represent the absorbance of the sample and blank treated cells, respectively.

### 2.7 Statistical analysis

The data were expressed as mean ± standard deviation (*SD*). Data were processed and analyzed using the SPSS version 22.0 (Windows, SPSS Inc.). *p* < .05 was considered indicative of statistically significant differences.

## 3 RESULTS AND DISCUSSION

### 3.1 Effect of SHA concentration on the stability of nanoparticles

**FIGURE 1:**
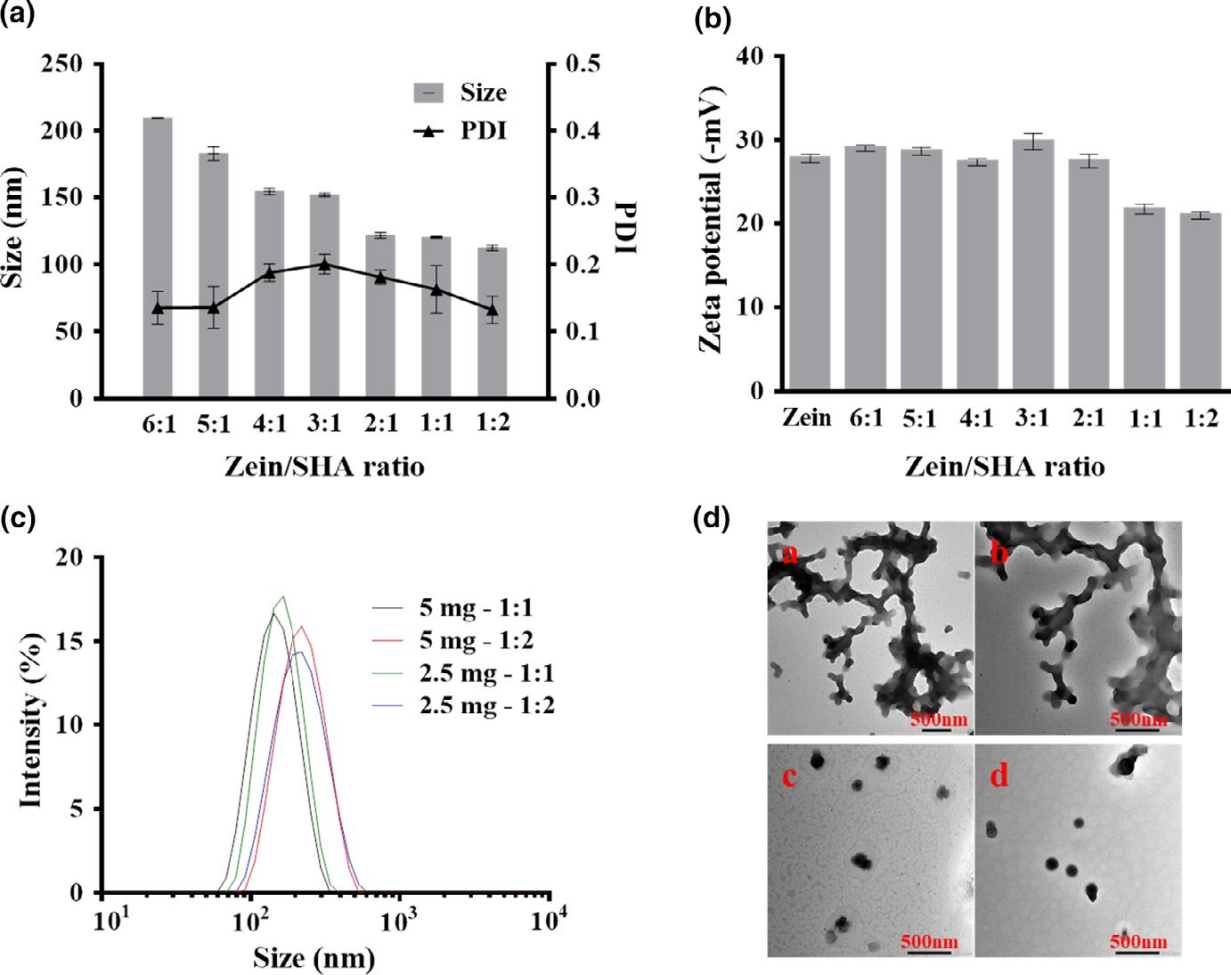
Effect of SHA concentration on the stability of nanoparticles (a) mean particle size and PDI of Zein‐SHA NPs (*n* = 3). (b) Zeta potential (*n* = 3) (c)and (d) the particle size distributions and TEM ((a) 5 mg‐1:1, (b) 5 mg‐1:2, (c) 2.5 mg‐1:1, (d) 2.5 mg‐1:2)

The isoelectric point of zein is 6.2, which is due to its unique amino acid ratio. These physicochemical properties are used to prepare core‐shell nanoparticles. Zein nanoparticles have a higher particle size when the SHA concentration is low, and are unstable and easy to aggregate. When the ratio of zein to SHA was 6:1, the particle size is 209.5 nm. As the relative content of SHA increased, the particle diameter formed was smaller and stable. When the ratio of zein to SHA was 1:1, the particle size was 112.3 nm. The PDI of nanoparticles with different SHA levels was 0.135–0.200, indicating that their particle size distribution is relatively narrow (Figure 1a). It shown that adding SHA can significantly reduce the size of nanoparticles and stabilize the nanocolloid solution. The most likely reason: as the amount of SHA increases, the particle size gradually becomes smaller, possibly because the degree of particle aggregation increases and the structure becomes tighter.

The result shown that with the increase of the cationic SHA concentration, the zeta potential changes from −29.83 to −20.93 mV (Figure 1b), which is related to the adsorption of cationic SHA molecules on the surface of anionic nanoparticles. This result is consistent with the formation of small, uniformly dispersed nanoparticles by electrostatic interaction. Different ratios of zein and SHA (zein [5 mg]:1:1, 1:2; zein [2.5 mg]:1:1, 1:2, w/w) were used to make Zein‐SHA with RES NPs in order to optimize the formulation of preparing nanoparticles. As shown in Figure 1c, the concentration of zein was 5 mg/ml, the ratio of zein to SHA was 1:1 and 1:2, through TEM (Figure 1d), it can be seen that the nanoparticles are unstable and aggregated, but when the concentration was 2.5 mg/ml the particle distribution is relatively dispersed. Although the particle size distribution measured by DLS is not much different, when observed by TEM, the zein concentration of 2.5 mg/ml was selected to prepare nanoparticles.

### 3.2 X‐ray diffraction analysis

**FIGURE 2:**
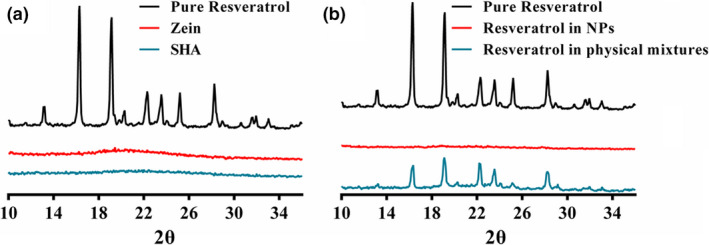
(a) XRD spectra of RES, Zein‐SHA with RES NPs, and RES was physically mixed with zein and SHA. (b) XRD spectra of SHA, RES and zein powder

The XRD patterns of the nanoparticles and their constituents were tested (Figure 2). It can be seen from Figure 2a that the 2θ diffraction peaks of resveratrol alone were 16.30, 19.15, 22.30, 23.55, 25.20, 28.25, which proved that resveratrol exists in the form of crystals. RES in nanoparticles did not show any characteristic intense sharp peaks, which shown that resveratrol was in amorphous form (Figure 2b).

### 3.3 Particle yield and RES‐loading efficiency

Encapsulation efficiency and particle yield affect the commercial application of nanocarriers. In order to pursue higher economic benefits and reduce waste, high encapsulation efficiency and particle yield are of great significance in delivery systems. Therefore, we explored the effect of adding different concentrations of resveratrol on the particle yield and the encapsulation efficiency of resveratrol. The results shown that both the particle yield and loading efficiency significantly decreased with increasing initial resveratrol concentration (Table 1).

**TABLE 1:** The particle yield, resveratrol‐loading efficiency, size and PDI of resveratrol‐loaded zein‐SHA nanoparticles

RES concentration (μg/ml)	Particle yield (%)	RES loading efficiency (%)	Size (nm)	PDI
100	95.41	66.50	172	0.126
200	93.68	68.60	165	0.111
300	93.27	62.52	152	0.102
400	91.35	54.26	163	0.114

### 3.4 Resveratrol release

**FIGURE 3:**
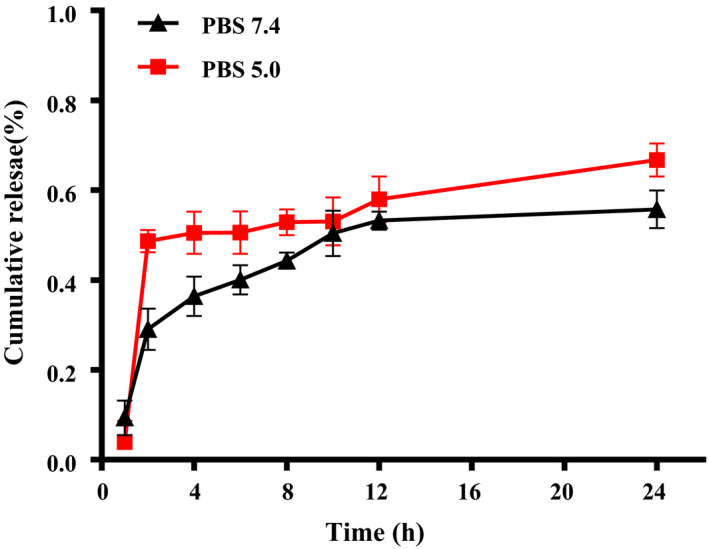
Cumulative RES release profiles Zein‐SHA with RES NPs at pH 7.4 and pH 5.0

The release of resveratrol in the nanoparticles was affected by pH conditions. RES released about 55.7% and 66.7% under the conditions of pH 7.4 and 5.0 at 24 hr (Figure 3). The possible reason was that under different pH conditions, the conformation of the nanocarrier has undergone different changes. At the time point of 2 hr, RES released 48.6% at pH 5.0, but at 7.4, the release was only 29.1%. The results shown that the nanoparticles encapsulating resveratrol have good acid‐responsive release characteristics, and were easier to release in the tumor microenvironment (pH = 5.0) than under normal physiological conditions (pH = 7.4).

### 3.5 Stability studies

**FIGURE 4:**
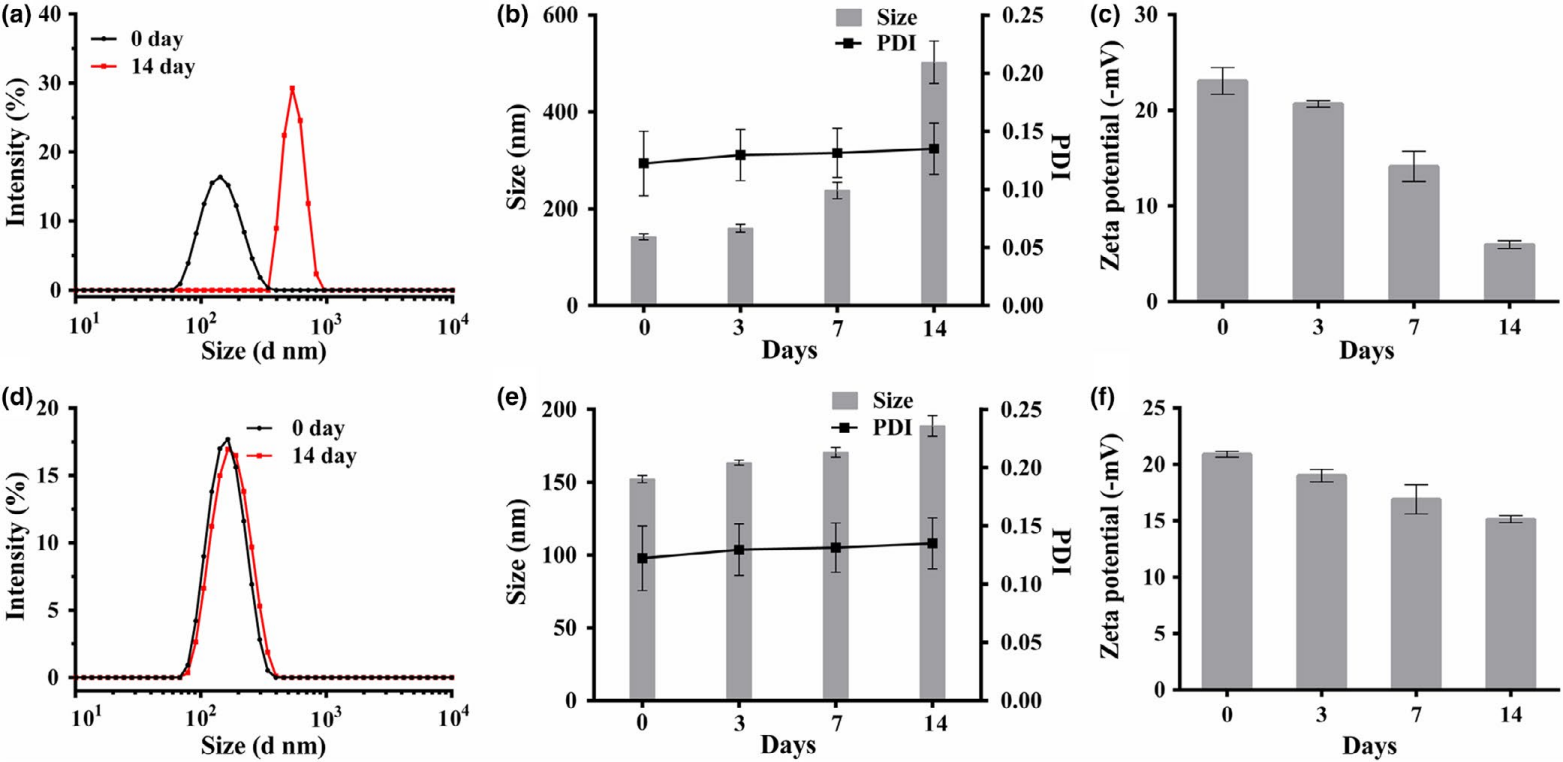
Particle size distribution of Empty NPs (a), Zein‐SHA with RES NPs (d); Compared the change of particle size and PDI between 0day and 14days (b), Zein‐SHA with RES NPs (e); Zeta potential changes of Empty NPs (c), Zein‐SHA with RES NPs (f) during storage (*n* = 3)

Comparing the stability of Zein‐SHA nanocarriers and RES loaded Zein‐SHA NPs. The newly prepared nanoparticles have a relatively narrow particle size distribution. However, after 14 days under 25°C, the particle size of Zein‐SHA with RES NPs remains unchanged and had better stability (Figure 4d). The particle size of the empty carrier becomes larger and has a wider particle size distribution (Figure 4a). The PDI of the two kinds of nanoparticles did not change significantly (Figure 4b,e), but the potential of the empty carrier changed greatly (Figure 4c), while the Zein‐SHA with RES NPs changed less (Figure 4f), Shown that Zein‐SHA with RES NPs had good storage stability.

### 3.6 Antioxidant activities of encapsulated resveratrol

**FIGURE 5:**
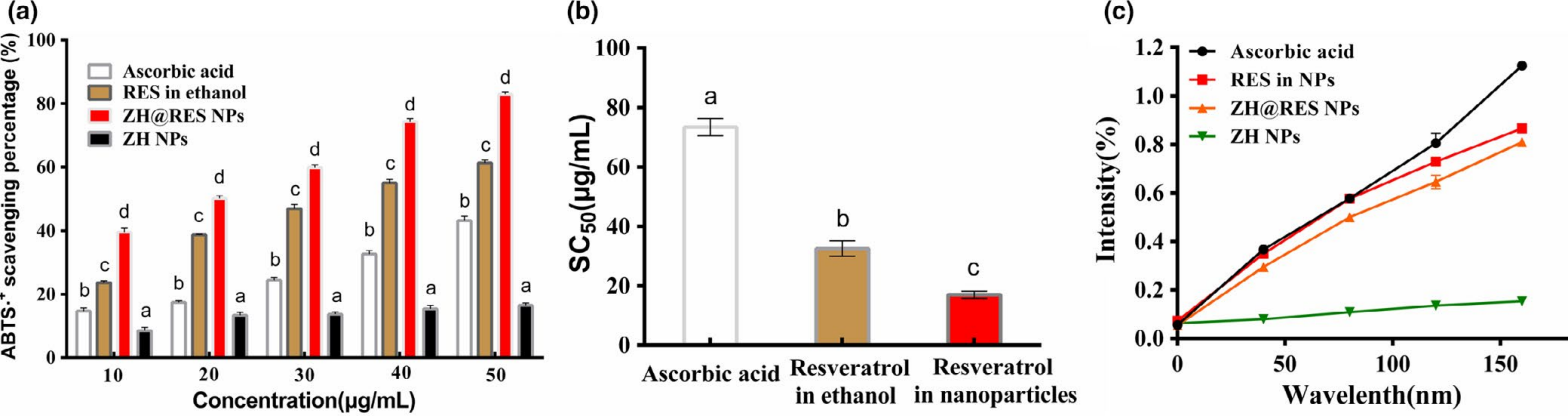
Compared the antioxidant capacity of Ac, RES, Zein‐SHA with RES NPs, Zein‐SHA NP. (a)ABTS^+^ scavenging activity; (b) SC_50_; (c) Reduction ability. Different letters indicate significant differences (*n* = 3, *p* < 0.05)

Resveratrol has good antioxidant activity. Based on this, the scavenging rate of ABTS free radicals and Fe^3+^ reduction ability of free and nanoencapsulated resveratrol were investigated. The results shown that Zein‐SHA with RES NPs had a significant difference in the scavenging rate of ABTS free radicals compared with RES and ascorbic acid (Ac; Figure 5a). The SC_50_ of Zein‐SHA with RES NPs, RES, and Ac are 16.97, 32.52 and 73.34 mg/ml, respectively (Figure 5b). The empty NPs exhibited little antioxidant properties, the scavenging activity value was around 16.47% when empty NPs concentration was 50 μg/ml, while Ac, RES, and Zein‐SHA with RES NPs scavenging activity value were around 43.19%, 61.35%, 82.95%. The reduction ability of Zein‐SHA with RES NPs, RES, and Ac was compared through the formation of Perl's Prussian blue. In addition, Zein‐SHA NPs were used as a negative control. The results shown that the reducing ability of Zein‐SHA with RES NPs was second only to Ac and higher than free RES, and Zein‐SHA NPs as a control group had little reducing power.

### 3.7 In vitro cytotoxicity

**FIGURE 6:**
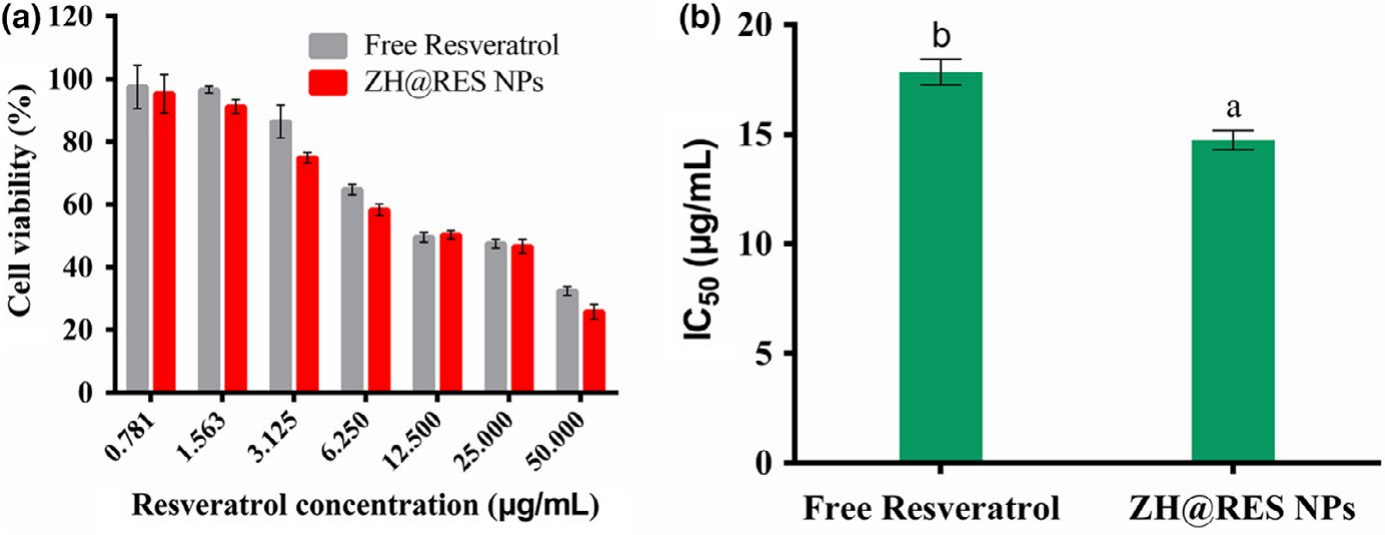
Zein‐SHA with RES NPs and RES antitumor effect (a) Cell viability of 4T1 cells (*n* = 3). (b) IC_50_ values (*n* = 3, *p* < 0.05)

Breast cancer is one of the killers threatening women's health (Freddie et al., 2018). The literature indicates great strides in early diagnosis and treatment of breast cancer. However, this cancer remains to be a public health issue. Investigating the antitumor effect of RES, the results of MTT shown that RES has a better inhibitory effect on the proliferation of 4T1 cells, and the inhibitory effect increased as the concentration of RES increased. When the RES concentration was 50 μg/ml, the cell survival rates of Zein‐SHA with RES NPs and RES were 32.39% and 25.77%, respectively (Figure 6a), and the IC_50_ was 17.84 and 14.73 μg/ml, respectively (Figure 6b). It shown that Zein‐SHA with RES NPs had a better inhibitory effect on 4T1 tumor cells.

## 4 CONCLUSIONS

The solubility of resveratrol limits its application in the food industry. The resveratrol‐loaded nanoparticles we developed can solve the problems of poor solubility and low bioavailability. The nanoparticles formed are relatively small, have a narrow particle size distribution, and have good antiaggregation stability in aqueous solutions. Moreover, they are able to efficiently incorporate resveratrol into their hydrophobic cores. Encapsulation of the resveratrol improved its water dispersibility, antioxidant activity and anticancer activity. Overall, our results suggest that the Zein‐SHA with RES NPs developed in this study may be highly effective nanocarriers for hydrophobic nutraceuticals.

## CONFLICT OF INTEREST

None of the authors are in conflict of interest with this research.

## AUTHOR CONTRIBUTIONS

**Qiankun Shi:** Data curation (lead); Formal analysis (lead); Investigation (equal); Validation (equal); Writing‐original draft (equal). **Xinya Wang:** Data curation (equal); Formal analysis (supporting); Validation (equal); Writing‐original draft (equal). **Xudong Tang:** Conceptualization (equal); Formal analysis (equal); Writing‐review & editing (equal). **Nuo Zhen:** Data curation (supporting); Formal analysis (supporting); Validation (supporting). **Yupeng Wang:** Data curation (supporting); Formal analysis (supporting); Writing‐original draft (supporting). **Zhijian Luo:** Formal analysis (equal); Validation (equal); Writing‐review & editing (supporting). **Hao Zhang:** Conceptualization (equal); Formal analysis (supporting); Funding acquisition (lead); Project administration (equal); Resources (equal); Writing‐review & editing (equal). **Jingsheng Liu:** Funding acquisition (supporting); Project administration (supporting); Resources (supporting). **Dongfang Zhou:** Funding acquisition (supporting); Methodology (supporting); Project administration (supporting); Writing‐review & editing (supporting). **Keke Huang:** Methodology (supporting); Resources (supporting).

## Data Availability

Research data are not shared.
